# Peanut lipids display potential adjuvanticity by triggering a pro‐inflammatory response in human keratinocytes

**DOI:** 10.1111/all.13475

**Published:** 2018-05-27

**Authors:** C. Palladino, M. S. Narzt, M. Bublin, M. Schreiner, P. Humeniuk, M. Gschwandtner, C. Hafner, W. Hemmer, K. Hoffmann‐Sommergruber, M. Mildner, O. Palomares, F. Gruber, H. Breiteneder

**Affiliations:** ^1^ Institute of Pathophysiology and Allergy Research Medical University of Vienna Vienna Austria; ^2^ Department of Dermatology Division of Biology and Pathobiology of the Skin Medical University of Vienna Vienna Austria; ^3^ Christian Doppler Laboratory for Biotechnology of Skin Aging Department of Dermatology Medical University of Vienna Vienna Austria; ^4^ Institute of Food Science University of Natural Resources and Life Sciences (BOKU) Vienna Austria; ^5^ Department of Dermatology University Hospital St. Poelten Karl Landsteiner University of Health Sciences St. Poelten Austria; ^6^ Karl Landsteiner Institute of Dermatological Research Karl Landsteiner Gesellschaft St. Poelten Austria; ^7^ Floridsdorf Allergy Center Vienna Austria; ^8^ Department of Biochemistry and Molecular Biology School of Chemistry Complutense University of Madrid Madrid Spain

To the Editor,

Currently, the earliest cellular and molecular signals driving allergic sensitization to peanuts are not fully understood, even though peanut allergens have been studied extensively. Meanwhile, lipids contained within allergen sources are emerging as players in the pathogenesis of allergies. Exposure of infants to peanut oil‐containing lotions was described as a risk factor for the development of peanut allergy.^1^ There is evidence that only peanut extracts containing lipids were able to induce sensitization.^2^, ^3^ How this occurs at the molecular level is still unknown. Moreover, in order to induce epicutaneous sensitization to purified peanut allergens, tape‐stripping of the skin is commonly performed.^4^ This procedure damages the skin and induces release of inflammatory cytokines in keratinocytes (KC).^5^

If crude peanut extract containing both allergens and lipids, but not purified peanut allergens alone, can sensitize mice via intact skin,^1^, ^3^ we wondered whether this effect might be due to the presence of peanut lipids (PNL). Therefore, we hypothesized that PNL might represent an adjuvant for peanut allergens in sensitization via the skin. To address this issue, we exposed human primary KC, major components of the outer layer of the skin, to PNL containing all major lipid classes (Figure S1) in the presence or absence of purified and functionally active peanut major allergens Ara h 1 or Ara h 2 (Figure S2), to study the induced immune response. Food‐processing modifications might contribute to influence the immune response; thus, roasted peanuts were used as source of PNL and allergens.

Details for materials and methods are provided in this article's online supporting information (Data S1).

First, we measured mRNA levels of IL‐8, IL‐6, TNF‐α, and IL‐1β in KC. We found that PNL, but not allergens alone, induced higher mRNA levels of these inflammatory mediators compared with the control (Figure 1). This effect persisted when PNL were coadministered with the allergens. Protein levels of IL‐8, IL‐6, and TNF‐α (Figure 2) correlated with the mRNA expression data, except for IL‐1β as no changes at protein levels were detectable compared with the control (data not shown).

**Figure 1:**
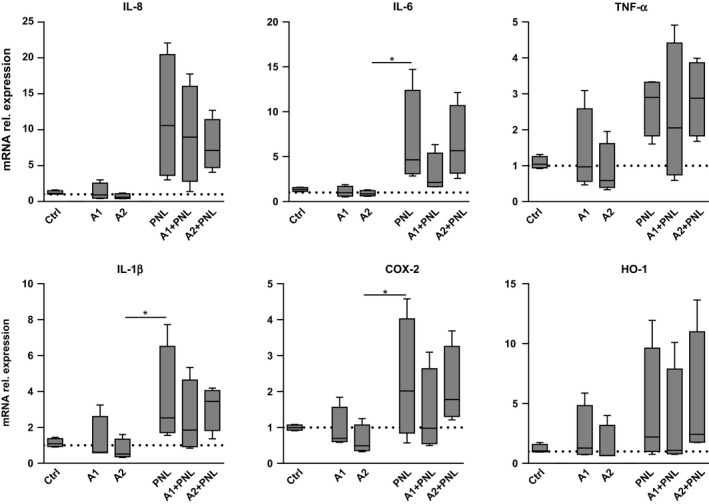
Transcriptional response of inflammatory genes in keratinocytes (KC) upon peanut lipids (PNL) and peanut allergens stimulation. Relative mRNA levels of IL‐8, IL‐6, TNF‐α, IL‐1β, COX‐2, and HO‐1 were quantified by real‐time PCR after 6 h of treatment with purified Ara h 1 (A1) or Ara h 2 (A2) in the presence or absence of PNL, and with PNL alone. Untreated cells: Ctrl. Results were obtained from 4 independent experiments. Values were normalized to the expression of beta‐2‐microglobulin. Significant differences were determined by ANOVA, followed by the Bonferroni post hoc test: *P < .05. Real‐time PCR primers are listed in Table S1.

**Figure 2:**
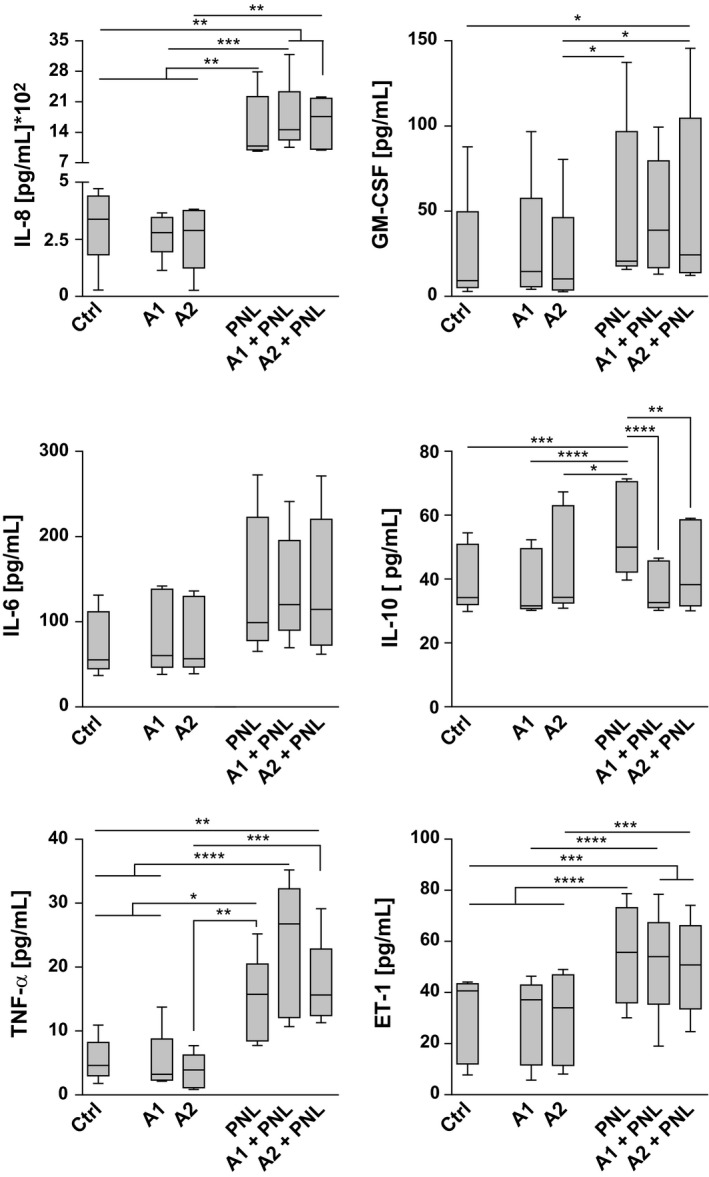
Capacity of peanut lipids (PNL) to induce the production of cytokines, chemokines, and mediators in keratinocytes (KC). Protein levels of IL‐8, IL‐6, TNF‐α, GM‐CSF, IL‐10, and ET‐1 in KC supernatants were measured upon 18 h of stimulation or 6 h for IL‐10 with purified Ara h 1 (A1) or Ara h 2 (A2) in the presence or absence of PNL, and with PNL alone. Untreated cells: Ctrl. Data were obtained from 5 independent experiments. Significant differences were determined by ANOVA, followed by the Bonferroni post hoc test: *P < .05, **P < .01, ***P < .001, ****P < .0001

Epithelial barrier cells respond to certain environmental stimuli with a set of cytokines able to activate dendritic cells to promote Th2 adaptive immunity.^6^ PNL, with or without allergens, induced in KC the release of significant amounts of GM‐CSF (Figure 2). Stimulation of KC with PNL for 6 hour triggered IL‐10 (Figure 2), a key tolerogenic, and anti‐inflammatory cytokine involved in the impairment of allergic inflammation. IL‐10 prevents the production of pro‐inflammatory cytokines by KC and the mobilization of Langerhans‐cells in the skin.^7^ Interestingly, the combination of PNL and Ara h 1 or Ara h 2 inhibited PNL‐induced IL‐10 release. Peanut allergens did not alter the capacity of PNL to induce pro‐inflammatory cytokines. However, via the inhibition of PNL‐induced IL‐10 release, they may contribute to extend the duration of inflammation and promote an environment suitable for the orchestration of Th2 immune responses leading to sensitization.

We also measured higher COX‐2 mRNA and protein levels in KC stimulated with PNL compared with the control (Figure 1; Figure S3). COX‐2 is essential for the induction of in vivo allergic inflammation in C57BL/6 mice^8^ and responsible for prostaglandin synthesis, inflammatory mediators with a key role in skin allergic inflammation. HO‐1 mRNA levels (Figure 1), but not proteins (data not shown), were also increased upon PNL treatment. HO‐1 is highly expressed in KC and is increased in response to stress and inflammatory stimuli.^9^ The capacity of PNL to activate human KC was further supported by our results demonstrating stress‐related morphological changes appearing after 24 hours of PNL treatment in the presence or absence of allergens (Figure S4) without affecting cell viability (Figure S5).

Endothelin‐1 (ET‐1), a peptide causing pruritus in mice and humans, is induced in KC in atopic dermatitis lesions and in response to house‐dust‐mite.^10^ PNL, but not allergens, enhanced the ET‐1 production (Figure 2), suggesting that PNL could promote itching and eventually skin barrier damage.

Our data demonstrated that KC were able to recognize PNL as exogenous stimuli that directly triggered the production of inflammatory mediators. Such mediators were shown to cause barrier disruption, facilitate allergen penetration and initiate cutaneous inflammation.^5^, ^6^ Interestingly, allergens coadministered with PNL were not able to modify (neither inhibit nor synergize) this effect of PNL, suggesting that the capacity of PNL to activate the innate immune system is maintained in the presence of the allergens, as in crude peanut extract.^2^, ^3^ In contrast, peanut allergens coadministered with PNL inhibited the production of PNL‐induced IL‐10, likely produced by KC as a compensatory mechanism. Altogether, this might indicate that PNL combined with allergens are necessary to prolong the pro‐inflammatory milieu induced by PNL which might contribute to favor allergic sensitization.

Here, we show that purified PNL activate KC and promote a pro‐inflammatory response similar to the effect of tape‐stripping,^5^ proposing a potential role for PNL as adjuvant for peanut allergens. Indeed, without the PNL‐induced pro‐inflammatory milieu, the allergens were unable to elicit any response in KC.

Our findings are in line with previous studies demonstrating that the matrix from allergen sources contains compounds that might aid allergic sensitization and lipids may play a fundamental role.^1^, ^2^, ^3^ The pro‐inflammatory mediators produced by KC upon PNL stimulation may perhaps lead to barrier disruption and promote the proper environment allowing Ara h 1 or Ara h 2 to penetrate the epidermis and recruit neighboring cells involved in the allergic sensitization process.^6^ Further studies are needed to fully elucidate the mechanisms involved in this response and whether a specific lipid component drives these effects.

In summary, we describe for the first time the impact of purified PNL on human primary KC. We have identified in PNL a potential adjuvant for peanut allergens, capable of directly activating KC, and inducing a pro‐inflammatory response which allergens might preserve via inhibition of PNL‐induced IL‐10 thus contributing to the duration of the inflammation. This might provide peanut allergens with the right milieu for the orchestration of Th2 immune responses leading to sensitization.

## ACKNOWLEDGMENTS

CP and PH were financially supported by the Austrian Science Fund (FWF) Doctoral Program MCCA W1248‐B30 and the Medical University of Vienna and KHS by the SFBF4603. MSN and FG were supported by the Federal Ministry of Science, Research, and Economy (BMWFW) of Austria and the National Foundation for Research, Technology, and Development of Austria to the Christian Doppler Laboratory for Biotechnology of Skin Aging.

## CONFLICT OF INTEREST

The authors declare that they have no conflicts of interest.
