# Supplementary figures and images for: Peanut lipids display potential adjuvanticity by triggering a pro‐inflammatory response in human keratinocytes
Source: Allergy. 2018 May 27;73(8):1746–9. doi: 10.1111/all.13475 (PMC6095042; doi:10.1111/all.13475)

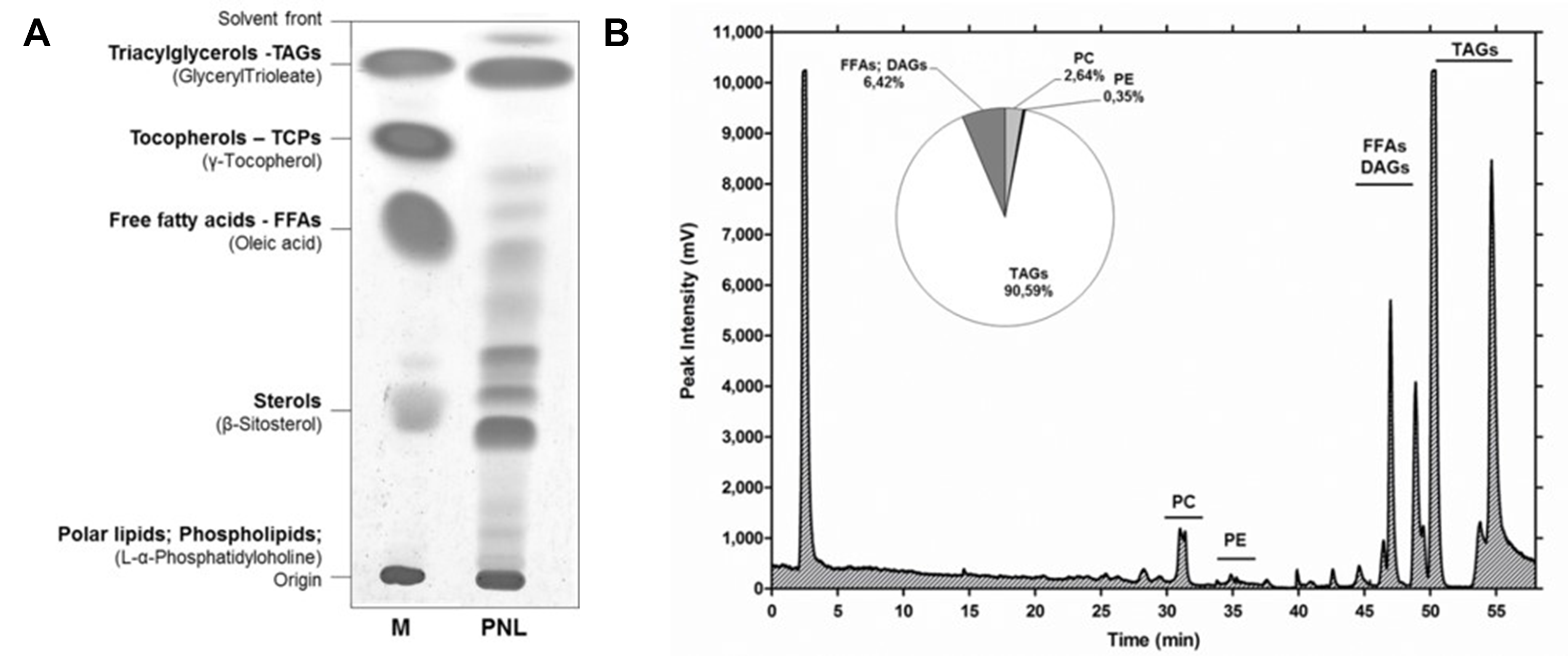

Supplement: Supplementary file 1 [file ALL-73-1746-s001.tif]

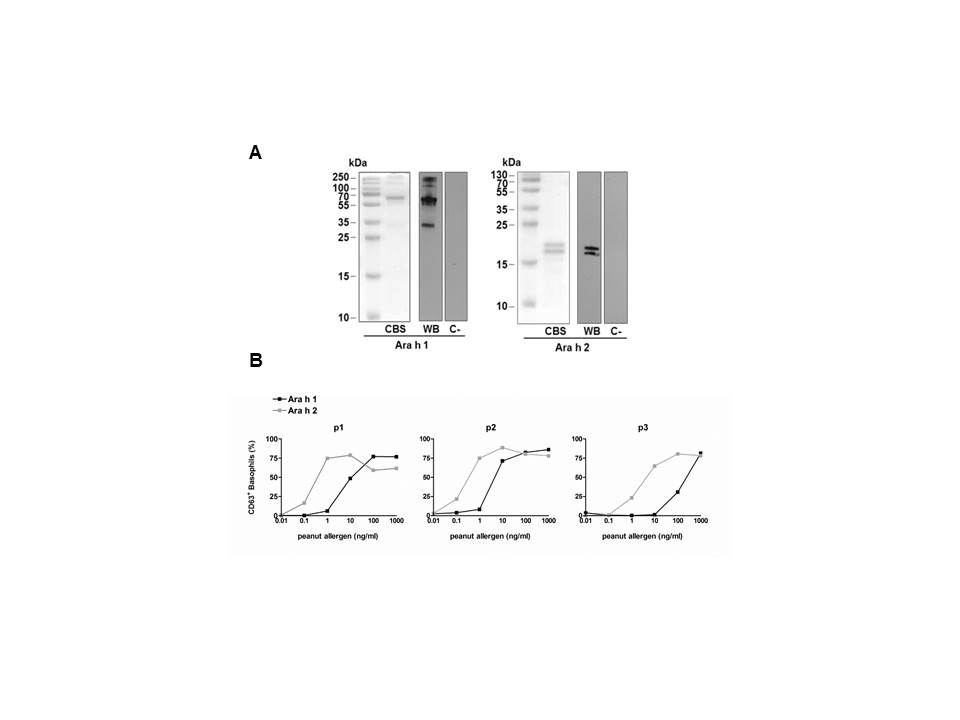

Supplement: Supplementary file 2 [file ALL-73-1746-s002.jpg]

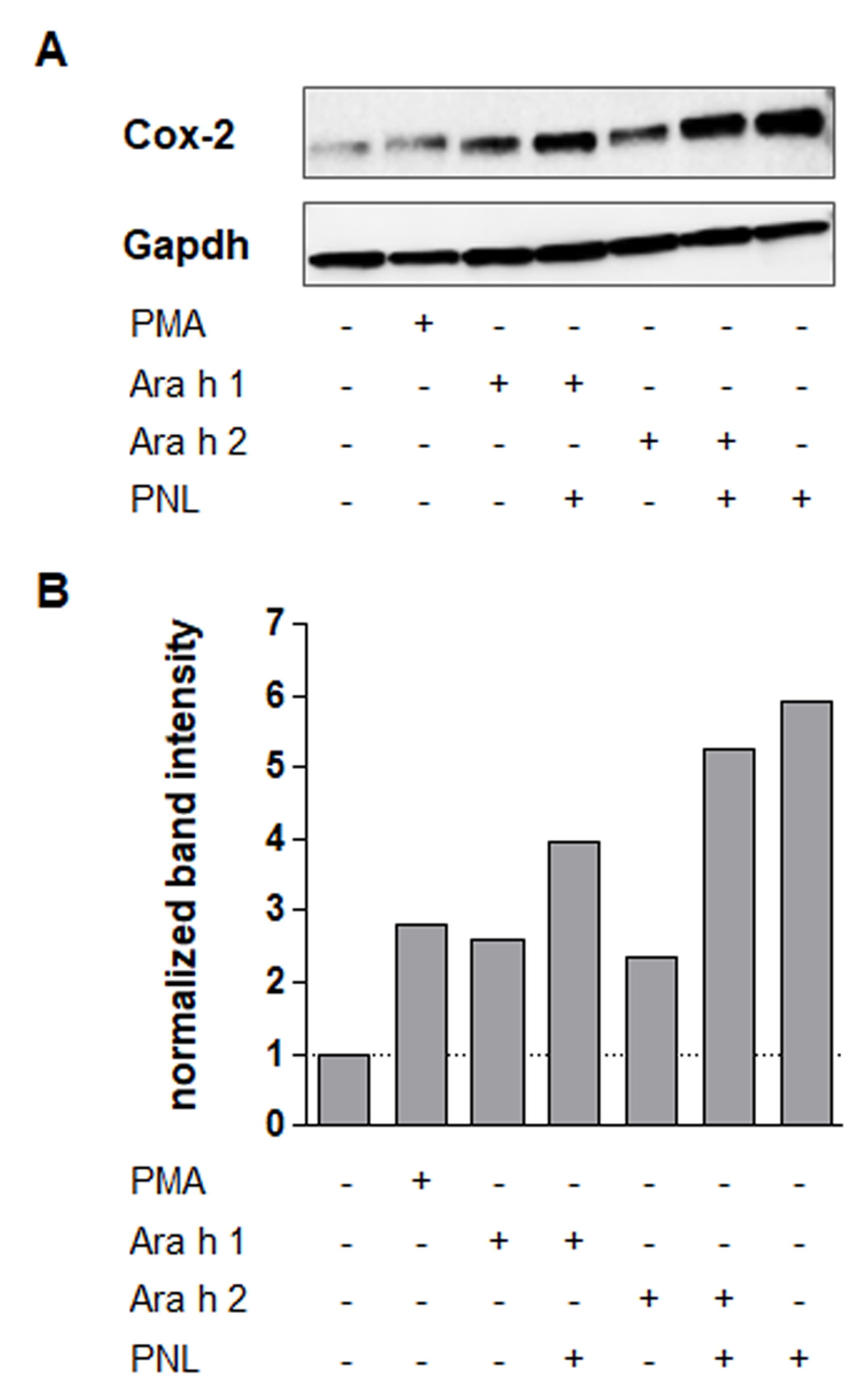

Supplement: Supplementary file 3 [file ALL-73-1746-s003.tif]

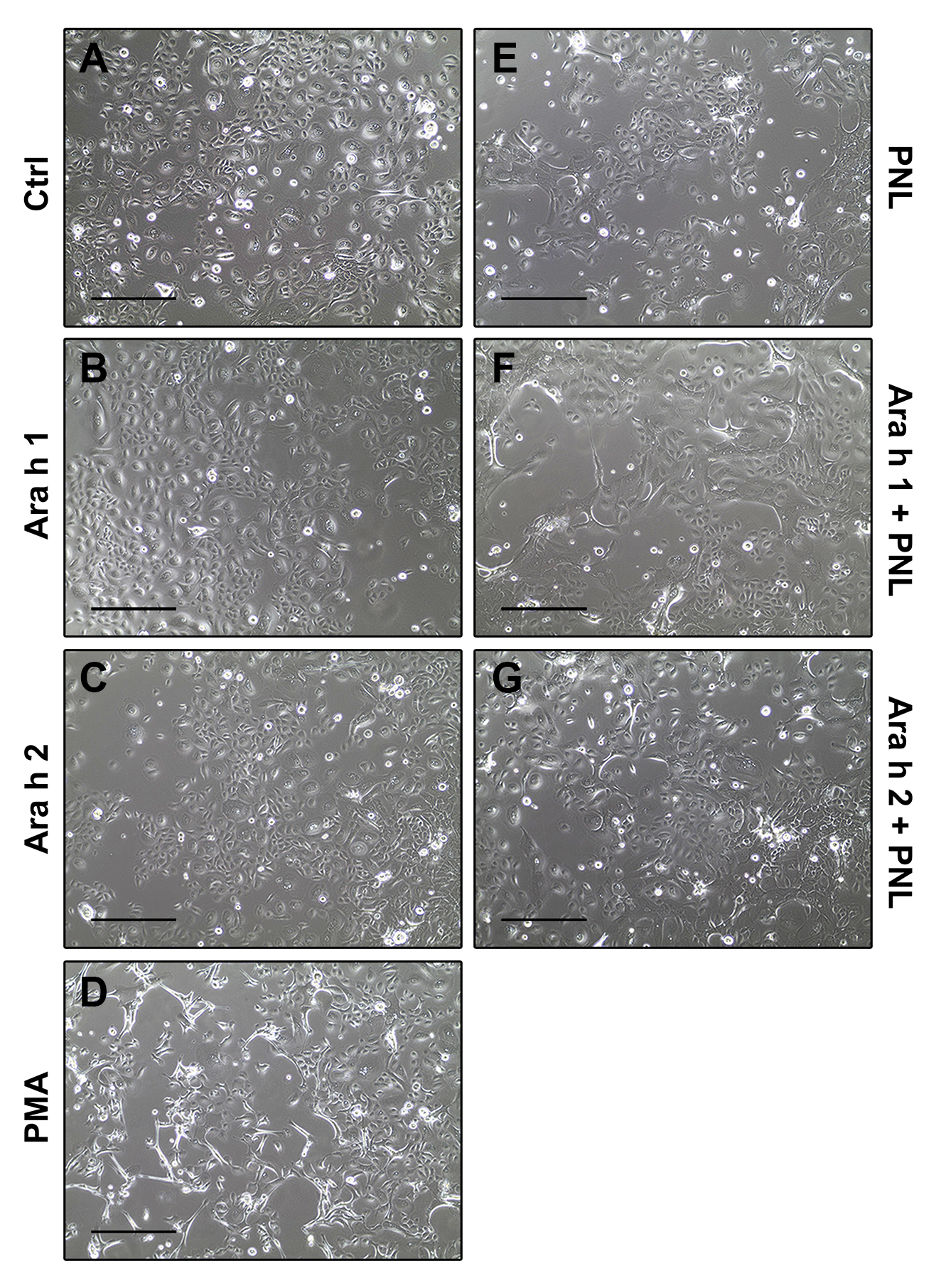

Supplement: Supplementary file 4 [file ALL-73-1746-s004.tif]

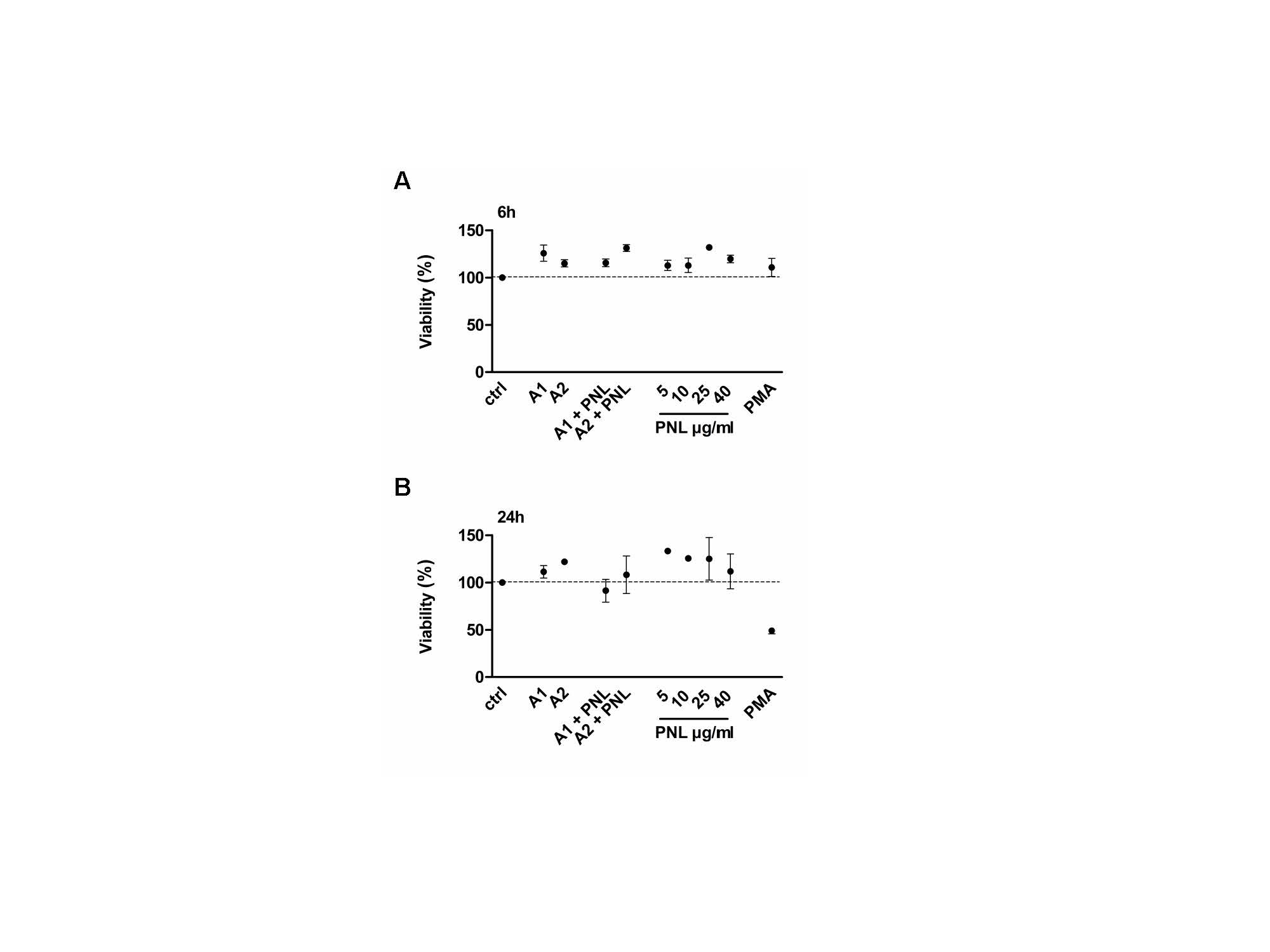

Supplement: Supplementary file 5 [file ALL-73-1746-s005.jpg]

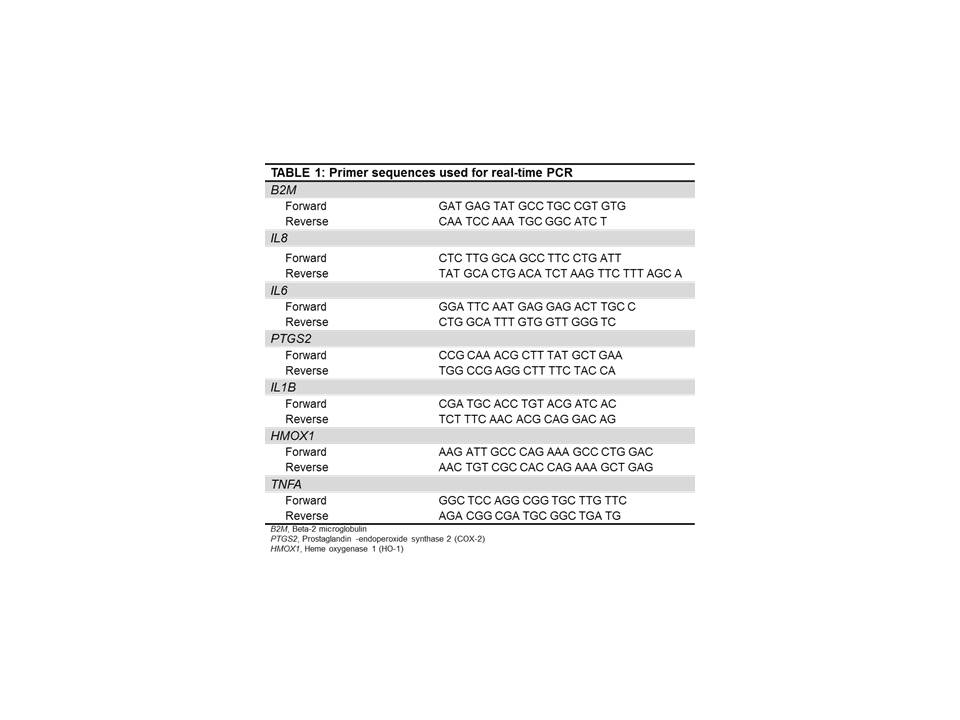

Supplement: Supplementary file 6 [file ALL-73-1746-s006.jpg]
