# Supplementary material for: Peanut lipids display potential adjuvanticity by triggering a pro‐inflammatory response in human keratinocytes
Source: Allergy. 2018 May 27;73(8):1746–9. doi: 10.1111/all.13475 (PMC6095042; doi:10.1111/all.13475)
Supplement: Supplementary file 7 [file ALL-73-1746-s007.docx]

**Peanut lipids display potential adjuvanticity by triggering a pro-inflammatory response in human keratinocytes**

C. Palladino^1^*, M. S. Narzt^2,3^*, M. Bublin^1^, M. Schreiner^4^, P. Humeniuk^1^, M. Gschwandtner^2^, C. Hafner^5,6^, W. Hemmer^7^, K. Hoffmann-Sommergruber^1^, M. Mildner^2^, O. Palomares^8^, F. Gruber^2,3^, H. Breiteneder^1^

*Both authors contributed equally.

^1^Institute of Pathophysiology and Allergy Research, Medical University of Vienna, Vienna, Austria

^2^Department of Dermatology, Division for Biology and Pathobiology of the Skin, Medical University of Vienna, Vienna, Austria

^3^Christian Doppler Laboratory for Biotechnology of Skin Aging, Department of Dermatology, Medical University of Vienna, Vienna, Austria

^4^Institute of Food Science, University of Natural Resources and Life Sciences (BOKU), Vienna, Austria

^5^Department of Dermatology, University Hospital St. Poelten, Karl Landsteiner University of Health Sciences, St. Poelten, Austria

^6^Karl Landsteiner Institute of Dermatological Research, Karl Landsteiner Gesellschaft, St. Poelten, Austria

^7^Floridsdorf Allergy Center, Vienna, Austria

^8^Department of Biochemistry and Molecular Biology, School of Chemistry, Complutense University of Madrid, Madrid, Spain

**Corresponding author:** Heimo Breiteneder, PhD

Institute of Pathophysiology and Allergy Research, Medical University of Vienna,

Waehringer Guertel 18-20, 1090 Vienna

Telephone: +43 (0)1 40400-51020

Fax: +43 (0)1 40400-51300

Email: heimo.breiteneder@meduniwien.ac.at

**Material and Methods**

*Isolation and characterization of peanut lipids (PNL)*

PNL were extracted from peanuts according to the method described by Folch *et al*. (1). The lipid composition of the extract was analyzed by thin-layer chromatography (TLC) for the major lipid classes using *n*-hexane/diethyl ether/acetic acid (70:29:1) as solvent system. In parallel, a further characterization was performed by reversed-phase (RP) HPLC analysis. One milligram of PNL dissolved in isopropanol was applied to a Phenomenex C18 column (100 x 3 mm; 2.6 µm particle size) with a mobile phase of H_2_O/acetonitrile = 1:1 with 0.2 % formic acid and a flow rate of 0.35 ml∙min^-1^. Detection was performed in an evaporative light scattering detector (ELSD). PNL resulted negative when tested for the presence of innate immune system contaminants by means of a cellular assay with the reporter cell lines THP1-XBlue and THP1-XBlue-MD2-CD14 (InvivoGen, San Diego, California, USA). Eventual traces of allergens in the PNL preparation were analyzed by basophil activation test (BAT). PNL preparation at any of the assayed concentrations (0.01, 0.1, 1, 10, 100, and 1000 ng/ml) did not trigger basophils activation in peanut allergic donors. PNL were used for experiments with human primary KC at a final concentration of 25 µg/ml, after 5 minutes of sonication in serum-free medium.

*Isolation and purification of Ara h 1 and Ara h 2*

Roasted peanuts were bought from a local supermarket. Crude peanut flour was prepared by grinding peanuts in a food blender to a fine powder. The powder was defatted three times with *n-*hexane (1:5 w/v), and then used for protein extraction. Ara h 1 and Ara h 2 were purified from peanuts as previously described (2), with minor modifications. Crude peanut extract was applied onto a ConA Sepharose-4B (GE Healthcare Bio-Sciences AB, Uppsala, Sweden) for purification of Ara h 1. Affinity chromatography fractions of partially purified Ara h 1 were pooled, concentrated by ultracentrifugation using an Amicon centrifugal filters with a 10 kDa MWCO membrane (Merck Millipore, Massachusetts, USA), and then applied to a HiPrep 26/60 Sephacryl-S200 High Resolution (GE Healthcare Bio-Sciences AB, Uppsala, Sweden) attached to a ÄKTA FPLC system (GE Healthcare Bio-Sciences AB, Uppsala, Sweden). Only size exclusion fractions of pure protein were pooled. For the purification of natural Ara h 2, crude peanut extract was applied to a HiPrep 26/60 Sephacryl-S200 High Resolution (GE Healthcare Bio-Sciences AB, Uppsala, Sweden) attached to a ÄKTA FPLC system (GE Healthcare Bio-Sciences AB, Uppsala, Sweden). Only size exclusion fractions with proteins of 10-25 kDa size were pooled, and loaded to a Q-Sepharose Fast Flow anion exchange column (GE Healthcare Bio-Sciences AB, Uppsala, Sweden) attached to a ÄKTA FPLC system (GE Healthcare Bio-Sciences AB, Uppsala, Sweden). The column was equilibrated with 20 mM Tris/HCl pH 8, and eluted with a 0-30% gradient of 20 mM Tris/HCl pH 8, 1 M NaCl. Ion-exchange fractions were dialyzed against water and concentrated by ultrafiltration in an Amicon spin column with a 3 kDa MWCO membrane (Merck Millipore, Massachusetts, USA). These fractions were loaded onto a preparative RP-HPLC Jupiter 4 μm Proteo 90Å, LC column (Phenomenex, Torrance, CA, USA) attached to an UltiMate 3000 Pump (Dionex, Sunnyvale, CA, USA). Bound proteins were eluted with a gradient from 10-70% of 0.9% trifluor-acetic acid (TFA) in acetonitrile (ACN). Peaks eluting at 22 minutes were shown to be enriched in Ara h 2. The identity of the purified allergens was confirmed by Western blot (Fig. S2 A) performed with anti-Ara h 1 (MA-2F7) and anti-Ara h 2 (MA1-4C) antibodies purchased from Indoor Biotechnology, Cardiff, UK. Endotoxin contamination of both peanut allergens was removed by EndoTrap columns (Hyglos GmbH, Bernried am Starnberger See, Germany). Remaining endotoxin measured by the LAL test (EndoZyme, Hyglos GmbH, Bernried am Starnberger See, Germany) was below 10 EU/mg. Both peanut allergens were negative when tested for innate immune components contaminants by a cellular assay with the reporter cell lines THP1-XBlue and THP1-XBlue-MD2-CD14 (InvivoGen, San Diego, California, USA). For the experiments with KC, Ara h 1 and Ara h 2 were used at a concentration of 25 µg/ml.

*Ethical Approval*

The study was approved by the Ethics Committee of the Medical University of Vienna (1149/2011) and the Ethics Committee of Lower Austria (GS1-EK-4/242-2013). Written informed consent was obtained from all subjects.

*Basophil activation test (BAT) assay*

Ara h 1 and Ara h 2 were tested in BAT as functional assay (3). Heparinized whole blood from 3 peanut allergic donors was incubated for 15 minutes at 37°C with titrated concentrations of Ara h 1 or Ara h 2 (0.01, 0.1, 1, 10, 100, and 1000 ng/ml). Basophils were then gated for CCR3 (eBioscence, Santa Clara, California, USA) and CD123 (Biolegend, San Diego, California, USA). Basophil activation was presented as percentage of CD63^+^ cells (4). See Fig. S2.B.

*Cell culture*

Primary human epidermal KC were prepared from non-allergic adult donor’s abdominal skin obtained from plastic surgery. Freshly isolated cells were cultured and expanded by passaging twice in serum-free KC growth medium (KGM-2, Lonza, Basel, Switzerland) at 37°C and 5% CO_2_ before treatment. The interval between isolation and experiment was usually 9 days.

*Quantitative real-time PCR*

KC cultured in 12-well plates were treated when 80% confluent for 6 h with PNL and/or Ara h 1 or Ara h 2. Total RNA was extracted with the RNeasy 96 kit (Qiagen, Hilden, Germany) according to the manufacturer´s instruction. cDNA was synthesized with the iScript cDNA Synthesis Kit (Bio-Rad, Hercules, CA) and real-time PCR of *IL8, IL6, IL1B, TNFA*, *HMOX1 (HO-1)*, and *PTGS2* (*COX-2)* was performed in a LightCycler 480 (Roche, Basel, Switzerland). The target genes´ relative expression levels were calculated using beta-2 microglobulin (*B2M*) as a reference according to the model previously described (5) and normalized to the untreated control. Primer sequences for the target genes used are listed in the Table 1 in the supporting information.

*Quantification of released mediators in KC supernatant*

KC were cultured in 12-well plates and when 80% confluent stimulated with peanut allergens and/or PNL. After 18 h of treatment, and 6 h for IL-10 based on published data, supernatants were harvested and concentrations of the released cytokines and mediators were determined by the Luminex 200 System (Merck Millipore, Massachusetts, USA) or ELISA (BD Bioscience, San Diego, CA, USA). We have measured the amounts of the following cyto-/chemokines and mediators in the supernatants of KC upon treatments with allergens alone, PNL alone or in association with Ara h 1 or Ara h 2: IL-1β, IL-6, IL-8, GM-CSF, IL-10, CCL-2, IL-33, IL-25, TSLP, and ET-1. These treatments caused changes in the levels of the following cytokines: IL-8, GM-CSF, ET-1, TNF-α, IL-10, and IL-6. IL-8 and GM-CSF were measured with the HCYTOMAG-60K kit (Merck Millipore, Massachusetts, USA), ET-1 was measured with the HAGP1MAG-12 kit, TNF-α measured with the HTH17-MAG-14 kit, both purchased from Merck Millipore, Massachusetts, USA. Changes were observed also for IL-10 and IL-6, which were quantified by ELISA according to the manufactures’ instructions (BD Bioscience, San Diego, CA, USA).

*Western Blot*

Cultured human KC were treated when 80% confluent for 24 hours with PNL, alone and in presence of Ara h 1 or Ara h 2, with the allergens alone, and with phorbol-12-myristate-13-acetate (PMA). After the treatment KC were washed twice with PBS and then harvested with lysis buffer (70 mM Tris-HCl, pH 6.8, 1.1% SDS, 11.1% (v/v) glycerol, 0.005% bromophenol blue (BioRad Laboratories, Hercules, CA, USA), containing protease inhibitor cocktail (Abcam, Cambridge, UK) and Pierce Phosphatase Inhibitor Mini Tablets (Thermo Fisher Scientific, Massachusetts, USA). Samples of cell lysate were maintained on ice, sonicated for 20 seconds, and then centrifuged prior separation through a 4-15% gradient polyacrylamide gel under reducing conditions (Biorad laboratories, California, USA). Detection of COX-2 and GAPDH was performed by immunoblotting with a COX-2 antibody (ab15191, Abcam, Cambridge, UK) and a GAPDH antibody (5G4; HyTest, Turku, Finland), respectively. As secondary antibodies, goat anti-rabbit IgG-HRP (Biorad Laboratories, Hercules, CA, USA) or sheep anti-mouse IgG-HRP (GE Healthcare, Little Chalfont, UK) were used and chemiluminescent quantification on a ChemiDoc imager (Biorad Laboratories, Hercules, CA, USA) was performed. Protein band intensities (Fig. S3) were normalized to GAPDH by Image Lab 4.1 analysis software (BioRad Laboratories, Hercules, CA, USA).

*Viability assay and light microscopy of keratinocytes*

Cell viability was determined by MTT 3-(4,5-dimethylthiazol-2-yl)-2,5-diphenyltetrazolium bromide assay (Merck-Sigma-Aldrich, St. Louis, Missouri, USA). KC were prepared as described above, seeded into 96-wells plate, and treated for 6 and 24 h with peanut allergens at 25 µg/ml, with PMA at 10 ng/ml, and peanut lipids at 5, 10, 25 and 40 µg/ml. After incubation at 37°C for the indicated time points, the MTT assay was performed and absorbance read at 560 nm (Fig. S5). For morphological analysis KC were cultured as described above, plated into 12-well plates and when 80% confluent treated with lipids and/or allergens for 24 h. Images were taken using X10 objectives of an EVOS XL microscope (Life Technologies, California, USA).

*Statistical analysis*

Statistical analysis was performed using the GraphPad Prism software version 6 (GraphPad Software Inc., San Diego, CA, USA). Statistical significance was calculated using one-way ANOVA, followed by the Bonferroni post-hoc test. A p-value below 0.05 was regarded as significant: *p<0.05; **p<0.01; ***p<0.001; ****p<0.0001.

**References**

1. Folch J, Lees M, Sloane Stanley GH. A simple method for the isolation and purification of total lipides from animal tissues. *J Biol Chem* 1957;**226**:497–509.

2. Marsh J, Rigby N, Wellner K, Reese G, Knulst A, Akkerdaas J et al. Purification and characterisation of a panel of peanut allergens suitable for use in allergy diagnosis. *Mol Nutr Food Res* 2008;**52**:S272–S258.

3. Koppelman SJ, Wensing M, Ertmann M, Knulst AC, Knol EF. Relevance of Ara h1, Ara h2 and Ara h3 in peanut-allergic patients, as determined by immunoglobulin E Western blotting, basophil-histamine release and intracutaneous testing: Ara h2 is the most important peanut allergen. *Clin Exp Allergy* 2004;**34**:583–590.

4. Knol EF, Mul FP, Jansen H, Calafat J, Roos D, Clevers H. Monitoring human basophil activation via CD63 monoclonal antibody 435. *J Allergy Clin Immunol* 1991;**88**:328–338.

5. Pfaffl MW. A new mathematical model for relative quantification in real-time RT-PCR. *Nucleic Acids Res* 2001;**29**:e45.

**Legends to Figures**

**Figure S1.** Characterization of peanut lipids (PNL). **A.** PNL were separated into major lipid classes by TLC with *n*-hexane/diethyl-ether/acetic acid (70:29:1) in parallel with a lipid marker (M). The lipid samples loaded as markers are given in brackets, the corresponding major lipids classes are indicated in bold. Starting from the solvent front the extract contains: triacylglycerols (TAGs), free fatty acids (FFAs), highly polar lipids and phospholipids close to the origin. **B.** Semi-quantitative analysis by HPLC shows the relative distribution of lipid classes in the extract revealing that TAGs represent 91%, FFAs and diacylglycerols (DAGs) constitute 6.42%, phosphatidylcholine (PC) 2.64%, and phosphatidylethanolamine (PE) 0.34%, as summarized by the pie-chart.

**Figure S2.** Peanut allergen characterization. **A.** Coomassie Blue Staining (CBS) and Western Blot (WB) of purified natural Ara h 1 (left panel) and Ara h 2 (right panel). Molecular mass markers are indicated in kDa. Negative control membrane: C-. **B.** Basophil activation is shown as percentage (%) of CD63^+^ cells. Whole blood from 3 peanut allergic donors (p1, p2, p3) was incubated for 15 minutes at 37°C with titrated concentrations (0.01, 0.1, 1, 10, 100, and 1000 ng/ml) of Ara h 1 (black line) or Ara h 2 (gray line).

**Figure S3.** Cox-2 protein levels and relative quantification. **A**. Cox-2 protein levels were assayed by Western blot after 24 hours of stimulation with peanut allergens Ara h 1 or Ara h 2, with peanut allergens in association with PNL, with PNL alone, and with phorbol-12-myristate-13-acetate (PMA). (-), untreated cells. **B**. Cox-2 protein band intensities were normalized to GAPDH.

**Figure S4.** Morphology of keratinocytes (KC). Evaluated by light microscopy after 24 h of treatment with **B.** Ara h 1 (A1), **C.** Ara h 2 (A2), **F.** and **G.** their combination with PNL; **E.** with PNL alone, and **D.** with phorbol-12-myristate-13-acetate (PMA); **A.** untreated cells. Images are from one representative donor. Scale bar: 400 µm. Stress-related morphological changes appeared after 24 hours of PNL-treatment in presence or absence of allergens. PNL-treated KC (**E, F, G**) presented an elongated morphology different to the round shape of cultured untreated cells (**A**).

**Figure S5.** Viability (%) of keratinocytes (KC) upon stimulation. **A.** For 6 h and **B.** for 24 h, with purified natural allergens Ara h 1 (A1) and Ara h 2 (A2) alone, combined with peanut lipids (PNL), with PNL alone (5, 10, 25, 40 µg/ml), and with phorbol-12-myristate-13-acetate (PMA) as positive control. Graphs represent the mean ± SEM of 2 independent experiments. Ctrl: untreated cells.
